# Assessing lung consolidation in goats using different ultrasonographic techniques

**DOI:** 10.1007/s11259-024-10458-1

**Published:** 2024-07-03

**Authors:** Giuliano Borriello, Flaminia Valentini, Giulia Cagnotti, Maria Teresa Capucchio, Sara Ferrini, Simona Zoppi, Antonio D’Angelo, Claudio Bellino

**Affiliations:** 1 Department of Veterinary Sciences, University of Turin, Largo Paolo Braccini, 2, Grugliasco (TO), 10095 Turin, Italy; 2 Istituto Zooprofilattico del Piemonte Liguria e Valle d’Aosta, 10154 Turin, Italy

**Keywords:** Goats, Ultrasound, Lung, Necropsy

## Abstract

Goats are often affected by respiratory diseases and, despite ultrasonography can assess lung consolidations in several species, it is rarely used in these animals. So, this study evaluated the effectiveness of on-farm lung ultrasonography in detecting lung consolidations on 27 goats. The goats, scheduled for slaughter, underwent complete clinical examinations and lung ultrasonography. For the latter, both sides of the thorax were divided in four quadrants and examined using convex and linear probes before and after shaving the hair. Each quadrant was classified based on presence/absence of lung consolidation and maximum consolidation’s depth (4-point scale: 0 healthy; **1** depth < 1 cm; **2** depth < 3 cm; **3** depth > 3 cm). The lungs were examined at necropsy, 66% of goats exhibited lung consolidations and sensitivity (83%-89%), specificity (100%), and κ coefficient values (0.67–0.72) were high with all techniques. An higher (p ≤ 0.01) percentage of class 1 lesions were found at necropsy compared to all the ultrasonographic techniques. All the ultrasonographic techniques effectively detected lung consolidation deeper than 1 cm. So, ultrasonography seems an effective tool for lung examination in goats with chronic pneumonia. The examination using the linear or the convex probes without shaving the hair could be a promising tool for the on-field diagnosis of pneumonia, although further research on larger sample sizes are necessary to validate these findings.

## Introduction

Goat farming is recognized as crucial for the rural economies of several countries due to the resilience of this species to harsh conditions. Nevertheless, health and productivity of these animals can be severely affected by respiratory diseases (Kumar et al. 2014; Sáadatnia et al. 2023). Lung consolidation, in particular, results from the absence of air in the alveoli and may arise from infections of various origin (i.e. bacterial, fungal, viral) (Lee et al. 2014; Tharwat and Al-Sobayil 2017) or it can be associated to non-infectious factors such as lung infarction or atelectasis (Sartori and Tombesi 2010). In large ruminants, lung ultrasonography is widely used for detecting consolidations due to its safety, for both animals and operators compared to other efficient techniques such as Computer tomography and radiography, and the portability of modern ultrasound machines (Ollivett and Buczinski 2016). Moreover, the ultrasonography of cranial thorax with the linear transrectal probe, already used for reproductive management, has been suggested as a means for quick pneumonia monitoring.

Regarding small ruminants, although several studies have shown that lungs can be successfully scanned in sheep (Cousens and Scott 2015), few studies have explored the capability of this technique for detecting lung consolidations in goats (Tharwat and Al-Sobayil 2017; Abdullah et al. 2023). Kowing the efficacy of lung ultrasonography in this species could enhance pratictioner’ diagnostic capability and reduce the time spent for the examination so, to fill this gap, we compared on-farm ultrasonographic findings in healthy goats and those affected by lung consolidations obtained with two different ultrasound probes.

## Material and methods

### Preliminary experiments

Since the ultrasound examination (US) is operator-dependent, two preliminary experiments were conducted to assess the reliability of the operator that performed the main experiment. Three raters with different levels of experience in lung ultrasonography (**Exp:** CB, more than 15 years of experience– **Adv:** GB, between 5–10 years of experience– **Int**: FV, less than 5 years of experience) blindly reviewed goats’ lung images (n:50, age: 2–6 years, mixed breed, with/without lung consolidation) obtained from the Veterinary Teaching Hospital (University of Turin, Department of Veterinary Science) database. The images were recorded with convex and linear transducers from animals underwent necropsy. Each rater evaluated the consolidation presence and maximum depth with a score (**0**:healthy; **1**:lesion depth < 1 cm; **2**:lesion depth < 3 cm; **3**:lesion depth** > **3 cm) also used in the main experiment (Berman et al. 2019). The same criteria for the evaluation of lung consolidations were used at necropsy by a pathologist as described in the main experiment. The agreement between the operators’ evaluations and between the operators and the necropsy results was assessed.

The second preliminary experiment consisted in the examination of live animals randomly chosen from a population of goats, mixed breed, above 2 years old, scheduled for slaughtering. Each subject enrolled was separately examined by two operators (**Adv, Int**) who performed clinical examinations and thoracic US. For the latter, lung areas (3rd to 12th intercostal spaces) were examined using a linear and a convex transducer, with and without the hair shaved as described in the main experiment. Each consolidation was located and classified by depth according to the methodology already reported. The lungs were submitted to postmortem examination by a pathologist and the findings were compared to the ultrasonographic results. Sensitivity, specificity, positive predictive values, negative predictive values of both operators were calculated. Furthermore, Cohen’s κ coefficient was calculated for inter-operator and operator-necropsy agreement for both preliminary studies.

### Main experiment

#### Animal selection and clinical procedure

The sample population was randomly selected from goat herds reared in northern Italy (Piedmont region) and scheduled for slaughter. Animals older than 2 years were included in the study, a complete clinical examination was performed and the body condition score (BCS) was assigned on a scale from 1 (Emaciated) to 5 (Obese) (Nagy and Pugh 2012). All procedures were performed by the **Adv** (GB) operator and abided to the good clinical practices (The European Agency for the Evaluation of Medicinal Product 2000) and the experiment was approved by the Ethical Animal Care and Use Committee, University of Turin (Protocol no 0004176-28/11/2022).

#### Lung ultrasound examination

US were performed by the **Adv** on manually restrained, not-sedated goats in quadrupedal stance, using an Esaote MyLab™ OneVet (Esaote SPA, Genova, Italy). Convex (1–8 MHz) and a gynecological linear transducer (5–10 MHz) were used before and after shaving both the lung areas (3rd to 12th intercostal spaces) with an electronic clipper (Farmclipper Akku2, Kerbl, Germany). Dirt and grease, if present, were removed using tap water and a solution of 70% isopropyl alcohol was applied as transducer agent.

The lung scans were performed placing the transducer head firmly perpendicular to the skin of each intercostal space and the vertical planes were examined by moving the transducer from dorsal to ventral. The pleura and the superficial lung parenchyma were examined in real-time B mode at a scan depth of 6–7 cm and a frequency of 7.5 MHz. If needed, the depth was set at 12–16 cm (Scott 2016). Four US techniques were conducted based on the transducers and the hair status, labeled as: convex (**Cx**) and linear (**Ln**); convex after hair shaving (**CxS**), linear after hair shaving (**LnS**). Each procedure was timed by an assistant operator.

To localize consolidations, each lung area was divided in four quadrants by two lines: one horizontal parallel to the floor, passing through the shoulder joint, and one vertical passing through the 5th intercostal space. The vertical line was based on lung ultrasonography described by Pravettoni (Pravettoni et al. 2021). So, eight quadrants per animal were obtained and labelled alphabetically on the right side from A to D, on the left from E to H. So, each side had craniodorsal (A-E), caudodorsal (B-F), cranioventral (C-G), caudoventral (D-H).

Each lesion was located and recorded in the database by an assistant operator.

Moreover, lung consolidations were classified by maximum depth, measured starting from the most superficial point of the parenchyma, and classified as described in the preliminary experiment (**0** healthy; **1** lesion depth < 1 cm; **2** lesion depth < 3 cm; **3** lesion depth** > **3 cm).

To minimize bias, a randomization process was performed. The US examinations began with the **Cx**, then the animal was marked before returning to its group. When the marked goat was randomly picked again from its group, the **Ln** was performed, the lung areas were shaved and the goat returned again to its group. Then, the same randomization scheme was used for **CxS** and **LnS**.

The goats were slaughtered after US and the lungs were submitted within 1 h to the necropsy room of the Department of Veterinary Science and postmortem examinations were carried out by a pathologist (MTC). The lungs were examined visually and by manual palpation; then the lungs were cut in 1 cm strip to assess the presence of deeper consolidations. The consolidations were located according to quadrant classification (A-H), classified on the 4-point scale and sampled. After necropsy, the consolidated lung samples were refrigerated at 4 °C and sent to the Istituto Zooprofilattico Sperimentale del Piemonte, Liguria e Val D’Aosta (IZS) laboratory within 12 h for bacteriological and virological investigations.

#### Statistical analysis

Statistical analysis was performed using R version 4.3.1 (R Project for Statistical Computing, R Core Team, 2022). Clinical variables and lung consolidations were analyzed by standard descriptive statistics; normality was assessed using the Shapiro-Wilk test. Data are expressed as numbers, percentage, median and range or mean ± standard deviation (SD). Sensitivity and specificity of the US were determined by comparing the results with the necropsy findings. Cohen’s κ coefficient was calculated to measure operator agreement in both preliminary studies and between the US and the necropsy findings. Differences in the percentage of lesion distribution were assessed using the McNemar test. The P value was set to < 0.05.

## Results

### Preliminary experiment

The review of images and video recordings performed by the three authors revealed that most of the lungs were affected by consolidations (**Exp:** 62%; **Adv:** 51%; **Int:** 60%; necroscopy: 60%). Substantial to almost perfect agreement between the operators was found regarding the presence (Range:0.70 – 0.84) and depth classification of lung lesions (Range: 0.70 – 0.87). Similarly, a substantial to almost perfect agreement was found between observers’ findings and the necropsy results.

In the second preliminary experiment, most goats examined (61% – 11/18) had lung consolidations and both operators (**Adv** and **Int)** obtained similar sensitivity (Range: 55%–68%) and specificity values (Range: 93% – 96%). Similar values were found also for positive predictive values (Range: 73% – 83%) and negative predictive values (Range: 89% – 91%) by both operators with all the techniques. The κ values between the operators were high for all scans (**Cx** 0.80; **Ln** 0.81; **CxS** 0.81; **LinS** 0.80), whereas the comparison with necropsy findings were classified between “moderate” and “good”.

### Main experiment

#### Sample population

The study sample was 27 goats, mostly female (25/27 – 92.6%), mixed breed. The median age was 5 years and the mean BCS was 2.9/5 (± 0.3), the clinical signs potentially related to respiratory disease detected are reported in Table 1.

**Table 1:** Clinical signs potentially associated with respiratory disease detected in the sample population

	Induced coughing	Spontaneous coughing	Nasal discharge	Ocular discharge	Abnormal lung sounds
Total	29.6% (8/27)	3.7% (1/27)	33.3% (9/27)	7.4% (2/27)	11.1% (3/27)
No consolidation	11.1% (1/9)	0% (0/9)	11.1% (1/9)	11.1% (1/9)	0% (0/9)
Consolidation	38.8% (7/18)	5.5% (1/18)	44.4% (8/18)	5.5% (1/18)	16.6% (3/18)

Total: entire sample population; No consolidation: goats without lung consolidations detected at necropsy; Consolidation: goats with lung consolidations detected at necropsy

The auscultation did not showed abnormality in all goats but 3 animals, in which abnormal lung sound (wheezes) at auscultation and severe pneumonia was found at US with all the probes and at necropsy. The manual restraining during procedures and shaving was well tolerated.

#### Ultrasound examination

Almost all the lesions detected were compatible with consolidation, only in five cases small abscesses were found within them. Moreover, in one goat a consolidation within liquid-fibrinous pleural effusion was detected during the ultrasound examination. High levels of sensitivity were found with all the techniques and the κ coefficient indicated substantial agreement between the operators and the necropsy findings (Table 2).

**Table 2:** Ultrasonographic techniques before and after hair shaving

Probe	Hair	Se	Sp	PPV	NPV	κ
Convex	Not shaved	83%	100%	100%	75%	0.71
	Shaved	89%	100%	100%	82%	0.72
Linear	Not shaved	83%	100%	82%	82%	0.67
	Shaved	83%	100%	100%	75%	0.72

Se: sensitivity; Sp: specificity; PPV: positive predictive value; NPV: negative predictive value, κ:Cohen’s kappa coefficient calculated between the US examination and the necropsy findings

There were no differences in the percentage of lesions classified by depth between the different US techniques. A higher percentage of lesions was classified as < 1 cm at postmortem examination compared to ultrasonography (Table 3).

**Table 3:** Percentage of consolidations detected at ultrasonography and necroscopy arranged by depth classification

Examination	Hair	0	1	2	3
Convex	Not shaved	84.2%	4.5%*	3.2%	8.1%
	Shaved	81.9%	5.6%*	3.7%	8.8%
Linear	Not shaved	83.3%	5.6%*	3.2%	7.9%
	Shaved	81.5%	5.6%*	3.7%	9.3%
Necropsy	-	76.4%	12.5%	2.8%	8.3%

0 = healthy; 1 = lesion depth < 1 cm; 2 = lesion depth < 3 cm; 3 = lesion depth > 3 cm; *:*p*  ≤  0.01 when compared to the necropsy results of the same column

Despite these differences, the k coefficient indicated substantial (**CxS**: 0.73) or almost perfect (**Cx:** 0.93**; Lin:** 0.85**; LinS:** 0.86) agreement between the operators. Each scan took less than 5 min, on average, to complete (**Cx** 04:18 ± 1:47; **CxS** 03:38 ± 1:05; **Ln** 03:45 ± 1:54; **LnS** 03:41 ± 01:17) and no significant differences were found between US techniques (*p* > 0.05).

#### Necropsy

Lung consolidations were detected in 18/27 (66%) goats at postmortem examination. All lesions were classified as chronic. Both the presence of small abscesses and the pleural effusion detected through ultrasonography were confirmed at necropsy. In both lungs the ventrocranial quadrants were the most often affected (C:24%—G:18%). The percentage of consolidations was significantly higher for quadrant C compared to quadrants A, D, F, H (Range: 6%—16%; *p* ≤ 0.05) and E (Range: 4%; *p* ≤ 0.01).

When classified by lesion depth, most of the quadrants (52.9%) of the affected animals had at least one superficial lesion (1: < 1 cm), while deeper lesions (> 3 cm) were noted in 35.3% of the quadrants. The most frequent microbial agents isolated was *Staphylococcus warneri* (9%), *Manneheimia Hemolitica* (7%), Small ruminant lentivirus (7%) and *Achromobacter spp.* (7%). No agents were isolated in 13% of the lung samples.

## Discussion

To the best of our knowledge this is the first study to compare the efficacy of different ultrasonographic techniques for detecting lung consolidations in goats. In the preliminary study, US images comparison showed good agreement between operators’ observations and necropsy findings. Since the comparison was made with US images retrieved from our Veterinary Teaching Hospital database, we could not evaluate the operators’ US techniques. However, according to Buczinski (Buczinski et al. 2018), image interpretation rather than the technique used for image acquisition is the main source of disagreement between raters and in the present study, the observations by the **Adv** and the **Int** were as reliable as those of the **Exp**.

Likewise, comparison of the US scans of live goats performed by two operators (**Adv** and **Int**) showed excellent levels of agreement. Previous studies noted similar results for the evaluation of lung consolidation in humans (Ellington et al. 2017) and calves (Buczinski et al. 2018), indicating that potential disagreement due to operator differences in lung US image acquisition and interpretation can be overcome with proper training.

Regarding the main experiment, finding few clinical signs of respiratory disease in goats is not surprising. Clinical descriptors of pneumonia such as respiratory pathologic sounds lack precision in sheep (Scott et al. 2010). Indeed, in this species, abnormal respiratory sounds could be detected in lung consolidation due to advanced cases of ovine pulmonary adenocarcinoma, but these findings did not consistently correspond with the lesions distribution (Scott et al. 2010). In our study, wheezes were detected only in goats with severe pneumonia whereas, in animals with less severe consolidation, no pathological sounds were assessed. So, we can hypothesize that the poor correlation between lung consolidation and clinical signs is shared by both species. However, more study on larger samples should be performed to completely understand these findings.

**Fig. 1:**
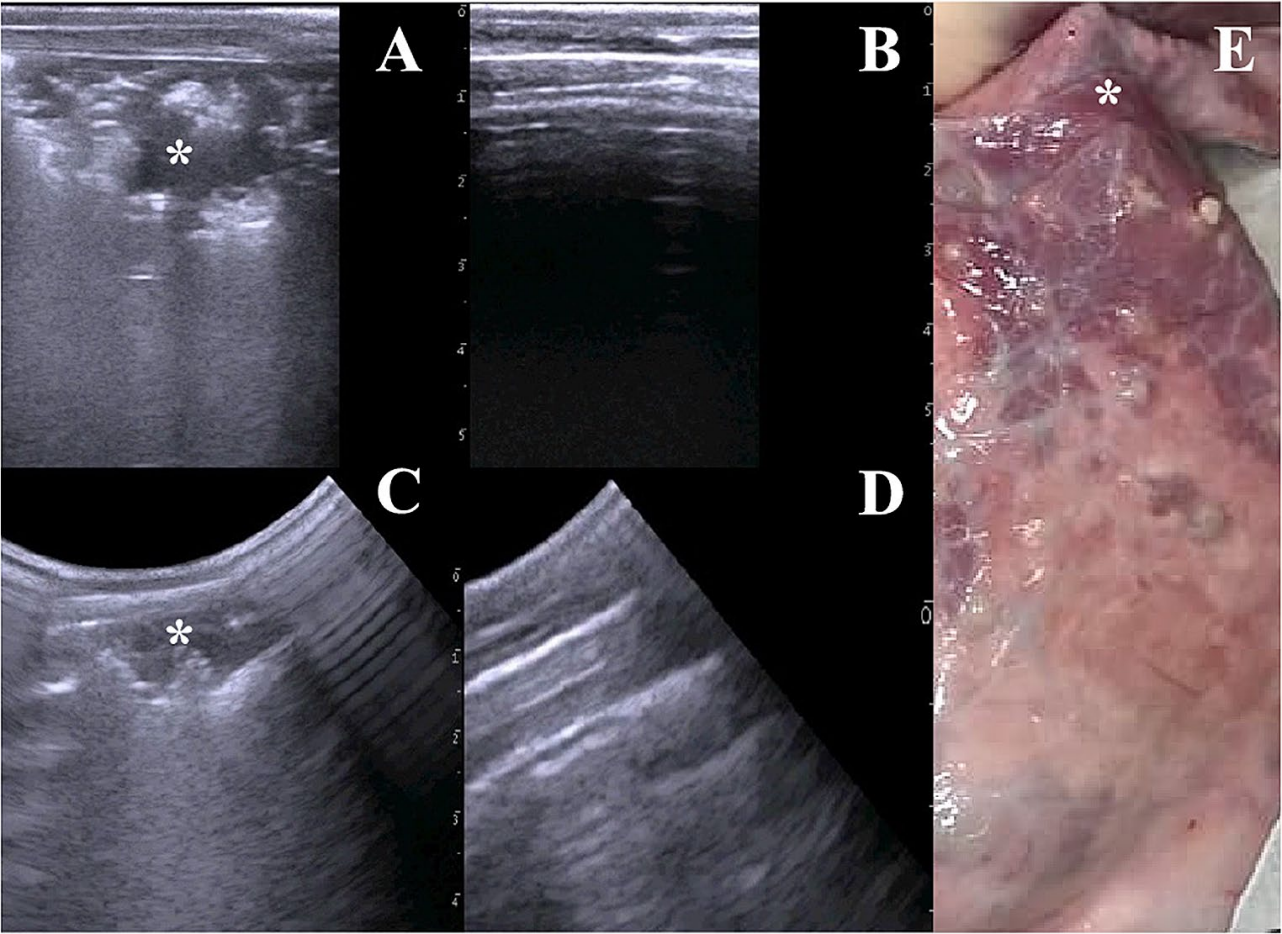
Images obtained during ultrasonographic examination with different transducers and necropsy. **A** Linear transducer, pathologic lung. **B** Linear transducer, healthy lung. **C** convex transducer, pathologic lung. **D** convex transducer, healthy lung. **E** Necropsy, pathologic lung *: lung consolidation

The high levels of sensitivity and specificity obtained with all US techniques, and the level of agreement with the necropsy findings suggest that ultrasonography is a reliable tool for the detection of chronic lung consolidation in goats (Fig. 1).

The agreement we found between imaging features and gross anatomy is shared by Tharwat et al. (Tharwat and Al-Sobayil 2017) and the levels of sensitivity and specificity are comparable to those reported in studies in bovine and sheep (Berman et al. 2019; Masset et al. 2022; Cousens et al. 2022). Additionaly, the efficacy of ultrasonography without shaving the area could be ascribed to short goat hair which, like that of calves (Buczinski et al. 2014), does not interfere with the examination. In sheep the wool thickness and cleanliness can affect the ultrasonography examination of several organs and in some cases shaving the wool could be necessary since air or dirt entrapped can reduce surface contact between the transducer and the skin (Scott et al. 2018; Borriello et al. 2021). While goat hair is usually thinner than sheep wool, we cannot exclude that, in curly-haired breeds such as Angora or in animals with dirty hair, the US procedure described here could be less efficient in detecting lung consolidations.

The depth classification disclosed a higher percentage of superficial consolidations (< 1 cm) not detected at US. Cousens et al. (2022) obtained similar results in sheep, comparing on-farm ultrasound for ovine pulmonary adenocarcinoma with necropsy findings. Despite focusing on a specific pathology, they shared our difficulties on detecting lesion smaller than 1 cm in lungs. So other studies are needed to determine whether this discrepancy is due to the suboptimal on-field environment for US or due to the goat anatomy. Consolidation depth is a recognized parameter for the prediction of disease severity in calves, however, superficial lesions seem to have minimal effects on their health (Berman et al. 2019). In this study, we cannot exclude that, due to differences in weight and size, lesions less than one centimeter deep may compromise a greater percentage of lung and significantly impact on goat health and productivity. Agreement between ultrasonography and necropsy findings was substantial or perfect for all techniques. These findings are particularly encouraging, since they suggest that chronic lung consolidation can be detected with both convex and linear transducer without shaving the hair, thus saving time during on-field screening. Few studies evaluated the efficacy of ultrasonography for the detection of lung lesions in goats, so comparing our findings with literature could be challenging due differences in equipment, methods, and study designs. In literature, studies involving goats have been conducted after shaving the lung areas (Tharwat and Al-Sobayil 2017; Xue et al. 2020; Abdullah et al. 2023), using microconvex (Tharwat and Al-Sobayil 2017; Xue et al. 2020) or linear probes (Abdullah et al. 2023) and gel as coupling agent. Tharwat and Al-Sobayil (2017) and Abdullah et al. (2023) described pneumonia-induced consolidation in goats without reporting the depth of the lesions, so data regarding the capability of detecting smaller consolidations were not provided. Xue et al. (2020), successfully detected lung consolidations with US in goat experimentally subjected to thoracic trauma, but the experimental design did not include the evaluation of consolidations ‘depth and anatomical distribution. Moreover, these studies were performed in experimental setting or in veterinary hospitals, so they did not explore the US diagnostic capability on field condition. Despite its efficacy, is important to note that this is not a flawless technique. Indeed, when a clear image of the deeper part of the parenchyma or the exact location of a lesion is necessary, it may be more advisable to rely on radiography or CT scans respectively, even though they are less suitable for on-farm examination (Castells et al. 2019). However, it must be considered that radiography can be affected by breathing and patient positioning, moreover the radiation emissions make it poorly suitable for flock screening. Regarding the CT, it is also affected by radiation issue and requires large equipment that is difficult to use in field conditions (Xue et al. 2020). Finally, while the microbial agents isolated from the lung samples were too heterogenous to draw any inferences, they are all potential causes of pneumonia and, given the chronic nature of the lesions, they are accidental or commensal microorganisms (Moroz et al. 2022).

So, ultrasonography seems an effective tool for lung examination in goats with chronic pneumonia, both **Cx** and **Ln** technique hold promise for lung consolidations’ diagnosing or screening in flocks. However, further studies on larger samples are needed to confirm the diagnostic capabilities of ultrasonography in goats and evaluate the effect of lesion depth on goat health.

## Data Availability

No datasets were generated or analysed during the current study.

## References

[CR1] Abdullah SM, El-Sheikh AKR, Mahmoud ARM, Attia NE (2023) Clinical, hematobiochemical and radiographical studies of caprine pneumonia. Slov Vet Res 60:65–74. 10.26873/SVR-1562-2022

[CR2] Berman J, Francoz D, Dufour S, Buczinski S (2019) Bayesian estimation of sensitivity and specificity of systematic thoracic ultrasound exam for diagnosis of bovine respiratory disease in pre-weaned calves. Prev Vet Med 162:38–45. 10.1016/J.PREVETMED.2018.10.025 30621897 10.1016/j.prevetmed.2018.10.025

[CR3] Borriello G, Guccione J, Di Loria A et al (2021) Fast focus ultrasound liver technique for the assessment of cystic echinococcosis in sheep. Animals 11:1–11. 10.3390/ani11020452 10.3390/ani11020452 PMC7914832 33572256

[CR4] Buczinski S, Forté G, Francoz D, Bélanger AM (2014) Comparison of thoracic auscultation, clinical score, and ultrasonography as indicators of bovine respiratory disease in preweaned dairy calves. J Vet Intern Med 28:234–242. 10.1111/jvim.12251 24236441 10.1111/jvim.12251 PMC4895545

[CR5] Buczinski S, Buathier C, Bélanger AM et al (2018) Inter-rater agreement and reliability of thoracic ultrasonographic findings in feedlot calves, with or without naturally occurring bronchopneumonia. J Vet Intern Med 32:1787–1792. 10.1111/jvim.15257 30133838 10.1111/jvim.15257 PMC6189347

[CR6] Castells E, Lacasta D, Climent M et al (2019) Diagnostic imaging techniques of the respiratory tract of sheep. 10.1016/j.smallrumres.2019.05.021

[CR7] Cousens C, Scott PR (2015) Assessment of transthoracic ultrasound diagnosis of ovine pulmonary adenocarcinoma in adult sheep. Vet Rec 177:366. 10.1136/vr.103298 26442526 10.1136/vr.103298

[CR8] Cousens C, Ewing DA, McKendrick IJ et al (2022) Efficacy of high-throughput transthoracic ultrasonographic screening for on-farm detection of ovine pulmonary adenocarcinoma. Vet Rec 191:e1797. 10.1002/VETR.1797 10.1002/vetr.1797 35788936

[CR9] Ellington LE, Gilman RH, Chavez MA et al (2017) Lung ultrasound as a diagnostic tool for radiographically-confirmed pneumonia in low resource settings. Respir Med 128:57–64. 10.1016/j.rmed.2017.05.007 28610670 10.1016/j.rmed.2017.05.007 PMC5480773

[CR10] Kumar A, Tikoo SK, Malik P, Kumar AT (2014) Respiratory diseases of small ruminants. Vet Med Int 2014. 10.1155/2014/373642 10.1155/2014/373642 PMC4293789 25610707

[CR11] Lee KS, Han J, Chung MP, Jeong YJ (2014) Consolidation. In: Radiology Illustrated: Chest Radiology. Springer-Verlag Berlin Heidelberg, pp 221–233

[CR12] Masset N, Assié S, Herman N et al (2022) Ultrasonography of the cranial part of the thorax is a quick and sensitive technique to detect lung consolidation in veal calves. Vet Med Sci 8:1229–1239. 10.1002/vms3.774 35218681 10.1002/vms3.774 PMC9122442

[CR13] Moroz A, Czopowicz M, Sobczak-Filipiak M et al (2022) The prevalence of histopathological features of pneumonia in goats with symptomatic caprine arthritis-encephalitis. Pathogens 11:1–16. 10.3390/pathogens11060629 10.3390/pathogens11060629 PMC9228274 35745483

[CR14] Nagy DW, Pugh DG (2012) Handling and examining sheep and gats. In: Pugh (ed) Sheep and Goat medicine, 2nd edn. Elsevier Sauders, Maryland Height, pp 2–3

[CR15] Ollivett TL, Buczinski S (2016) On-farm use of ultrasonography for bovine respiratory disease. Vet Clin North Am Food Anim Pract 32:19–35. 10.1016/J.CVFA.2015.09.001 26922110 10.1016/j.cvfa.2015.09.001

[CR16] Pravettoni D, Buczinski S, Sala G et al (2021) Short communication: diagnostic accuracy of focused lung ultrasonography as a rapid method for the diagnosis of respiratory disease in dairy calves. J Dairy Sci 104:4929–4935. 10.3168/jds.2020-19377 33663827 10.3168/jds.2020-19377

[CR17] Sáadatnia A, Mohammadi GR, Azizzadeh M et al (2023) Effect of ultrasonographic lung consolidation on health and growth in dairy calves: a longitudinal study. J Dairy Sci 106:8047–8059. 10.3168/JDS.2023-23296 37641278 10.3168/jds.2023-23296

[CR18] Sartori S, Tombesi P (2010) Emerging roles for transthoracic ultrasonography in pulmonary diseases. World J Radiol 2:203–214. 10.4329/wjr.v2.i6.203 21160632 10.4329/wjr.v2.i6.203 PMC2999323

[CR19] Scott P (2016) Practical use of ultrasound scan in small ruminant medicine and surgery. Vet Clin North Am Food Anim Pract 32:181–205. 10.1016/j.cvfa.2015.09.008 26922119 10.1016/j.cvfa.2015.09.008

[CR20] Scott P, Collie D, McGorum B, Sargison N (2010) Relationship between thoracic auscultation and lung pathology detected by ultrasonography in sheep. Vet J 186:53–57. 10.1016/j.tvjl.2009.07.020 19733102 10.1016/j.tvjl.2009.07.020

[CR21] Scott PR, Dagleish MP, Cousens C (2018) Development of superficial lung lesions monitored on farm by serial ultrasonographic examination in sheep with lesions confirmed as ovine pulmonary adenocarcinoma at necropsy. Ir Vet J 71:1–9. 10.1186/s13620-018-0134-0 30450192 10.1186/s13620-018-0134-0 PMC6219085

[CR22] Tharwat M, Al-Sobayil F (2017) Ultrasonographic findings in goats with contagious caprine pleuropneumonia caused by Mycoplasma capricolum subsp. capripneumoniae. BMC Vet Res 13:1–8. 10.1186/s12917-017-1167-4 28830505 10.1186/s12917-017-1167-4 PMC5568355

[CR23] The European Agency for the Evaluation of Medicinal Product (2000) Guideline on good clinical practices. In: ICH (ed) ICH E6 (R3) Guideline on good clinical practice (GCP), Amsterdam, pp 0–29

[CR24] Xue Y-Q, Wu C-S, Zhang H-C et al (2020) Value of lung ultrasound score for evaluation of blast lung injury in goats. Chin J Traumatol 23(1):38–44. 10.1016/j.cjtee.2019.11.005 10.1016/j.cjtee.2019.11.005 PMC7049640 32005413

